# Multiple and Bilateral Sporadic Renal Cell Carcinomas: A Surgical Challenge

**DOI:** 10.1155/2018/4325762

**Published:** 2018-12-20

**Authors:** Antonios Katsimantas, Spyridon Paparidis, Konstantinos Bouropoulos, Nikolaos Ferakis

**Affiliations:** Department of Urology, Korgialenio-Benakio Hellenic Red Cross Hospital, Athanasaki 1, 11526 Athens, Greece

## Abstract

Sporadic, synchronous, bilateral, or unilateral Renal Cell Carcinomas constitute a rare clinical entity. We report the case of a 68-year-old male patient who presented in our department due to incidentally discovered multiple, bilateral renal tumors. Magnetic Resonance Imaging demonstrated cT1b renal tumors at the lower pole of each kidney and a cT1a renal tumor at the upper pole of the right kidney. The patient underwent transperitoneal, laparoscopic left partial nephrectomy with renal artery occlusion, histology revealed high-grade, pT1b, clear-cell renal cell carcinoma; however we observed decline of patient's estimated glomerular filtration rate postoperatively. Forty days postoperatively, he underwent open partial nephrectomy for the right sided tumors with manual compression of the renal parenchyma and no use of ischemia. Histology revealed high-grade, pT1a, clear-cell renal cell carcinoma at the upper pole of the right kidney and low-grade, pT1b, clear-cell renal cell carcinoma at the lower pole of the right kidney. There was no additional decline in the serum creatinine value postoperatively. The patient avoided permanent or temporary dialysis and 6 months postoperatively he demonstrated no recurrence on imaging and his renal function remained stable.

## 1. Introduction

Sporadic, synchronous, bilateral, or unilateral Renal Cell Carcinomas (RCCs) constitute a rare clinical entity and a stressful challenge for the surgeon [1–5]. A single approach cannot be proposed as every case is almost unique [2]. Our aim is to present the management of an interesting case of sporadic, synchronous, bilateral, and multifocal RCC.

## 2. Case Report

In March 2018, a 68-year-old male patient was admitted to our department because of multiple renal tumors bilaterally, incidentally discovered during abdominal ultrasonography for screening purpose. His medical history included allergic reaction to iodinated contrast agent.

**Figure 1:**
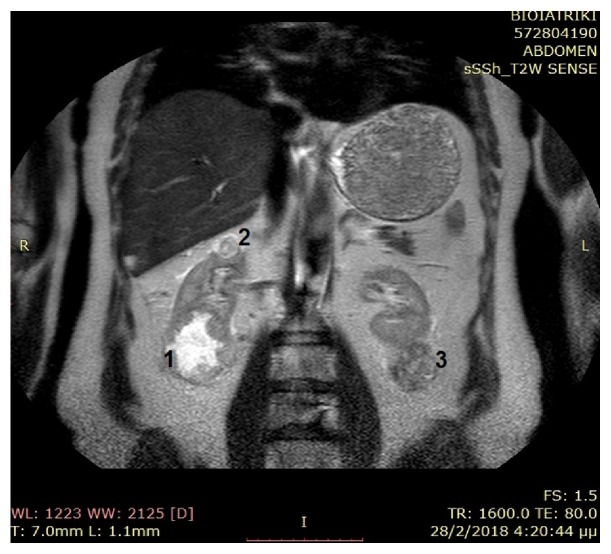
T2-weighted coronal MRI image demonstrating a 6.2x6.3 cm in size, solid mass at the lower pole of the right kidney (1), a 2.4 cm solid mass at the upper pole of the right kidney (2), and a 5.4x4.5 cm in size, solid mass at the lower pole of the left kidney (3).

Preoperative Magnetic Resonance Imaging (MRI) demonstrated the followings: (1) a 6.2x6.3 cm in size, solid mass at the lower pole of the right kidney, (2) a 2.4 cm solid mass at the upper pole of the right kidney, and (3) a 5.4x4.5 cm in size, solid mass at the lower pole of the left kidney (Figure 1). The lesions were exophytic and they demonstrated hyperintense signal on T2-weighted sequences and hypointense signal on T1-weighted sequences. All of them presented heterogeneous enhancement after intravenous administration of paramagnetic contrast agent. Blood chemistry levels were in the normal range. Preoperative serum creatinine value was 1 mg/dl (with normal values 0.6-1.2 mg/dl) and preoperative estimated glomerular filtration rate (eGFR) according to the Modification of Diet in Renal Disease (MDRD) formula was 78.98 ml/min/1.73m^2^.

**Figure 2:**
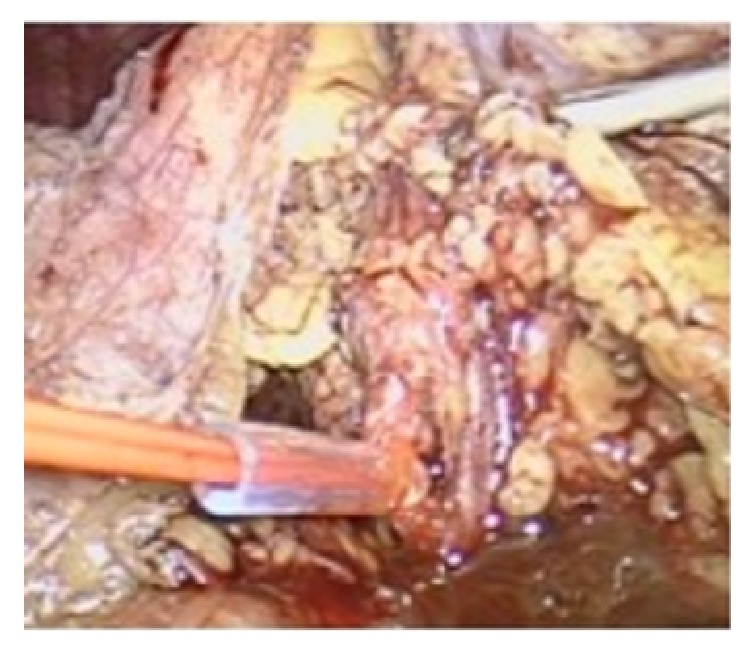
Intraoperative photo from the transperitoneal laparoscopic left PN demonstrating the occluded left renal artery using Rummel Tourniquet technique.

**Figure 3:**
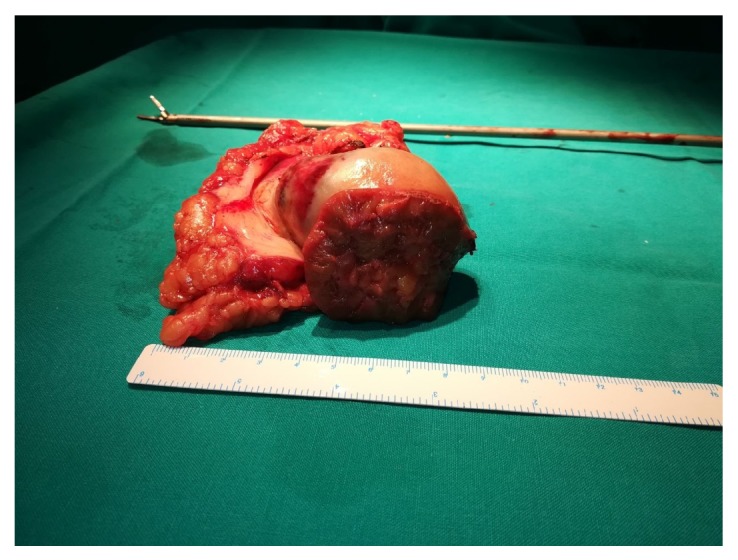
Postoperative photo demonstrating the surgical specimen of the left laparoscopic PN.

The patient underwent transperitoneal laparoscopic left partial nephrectomy (PN), which is the standard operative technique for cT1 renal tumors in our department, under general anesthesia. We decided to perform firstly PN on the left side to obtain histological information and to evaluate renal function before treating the right side. Standard operative technique in our department involves the application of warm ischemia (WI), by using a 2 mm, 30 cm vessel loop, a 2 cm cylinder sheath prepared from a 16 Fr Levin tube, and a large Hem-o-Lok clip (Rummel Tourniquet technique) in order to occlude renal artery (Figure 2). WI time was 24 minutes and the duration of the operation was 184 minutes. Postoperative course was complicated by fever and by an episode of atrial fibrillation (classified as Clavien grades 1 and 2, respectively, according to the modified Clavien scale). The patient was discharged on 5th postoperative day, the serum creatinine value was 1.8 mg/dl, and eGFR was 40.08 ml/min/1.73m^2^. Histology revealed clear-cell RCC, 5 cm in size, Fuhrman nuclear grade 3, and negative surgical margins (pT1b) (Figure 3).

**Figure 4:**
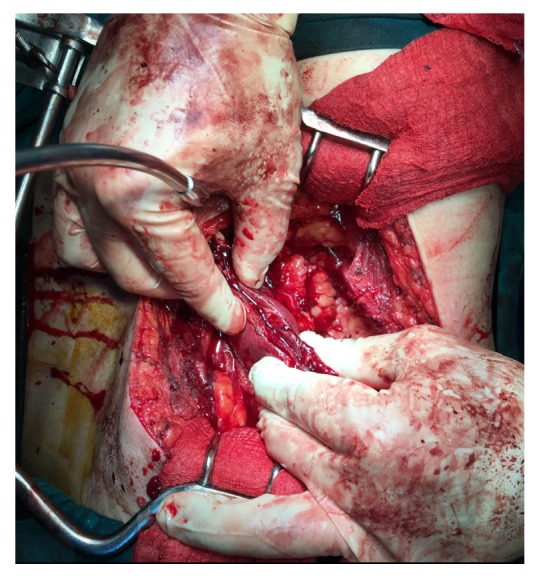
Intraoperative photo demonstrating manual compression of the renal parenchyma during the resection of the right renal tumor of the lower pole.

Forty days postoperatively, the patient was readmitted in order to be operated for the tumors of the right kidney. Serum creatinine value was 1.7 mg/dl and eGFR was 42.81 ml/min/1.73m^2^. The patient underwent open right PN under general anesthesia. We removed both of the polar tumors simultaneously. We did not apply any kind of ischemia in order to preserve renal function, but we just applied manual compression at the resection edges periodically in order to facilitate tumors' removal and completion of renorrhaphy (Figure 4). The patient had an uneventful postoperative course, his eGFR remained stable, and he was discharged on 5th postoperative day. Histology revealed clear-cell RCC, 2.2 cm in size, and Fuhrman nuclear grade 3 for the tumor of the upper pole (pT1a), with negative surgical margins and clear-cell RCC, 5.5 cm in size, and Fuhrman nuclear grade 2 for the tumor of the lower pole (pT1b). Focal microscopic invasion of the pseudocapsule was evident at the base of the low-grade tumor of the lower pole.

Six month postoperatively, the serum creatinine value was 1.44 mg/dl, eGFR was 51.85 ml/min/1.73m^2^, and there was no recurrence on MRI. The patient is scheduled for follow-up 12 months postoperatively.

## 3. Discussion

Bilateral RCC consist of a rare clinical entity accounting for 1-5% of patients with RCC [3]. Sporadic, synchronous, bilateral, or unilateral RCCs are even rarer, distinct categories of RCC, and their biological behavior is different from hereditary bilateral RCC [1, 3].

Nephron Sparing Surgery (NSS) is the treatment of choice for bilateral RCC, which can be accomplished by open, laparoscopic, or robot-assisted laparoscopic technique [2–5]. According to the literature, the surgical approach can be single or staged and depends on the number, location and size of renal tumors, as well as to the surgeon experience [2, 3, 5]. Therefore, detailed preoperative imaging is of paramount importance [4]. In general, the management of multiple, bilateral, and/or unilateral RCC constitutes a surgical challenge and there is not a single approach which can be proposed for all of the cases [1–3].

The main goals of PN are oncologic control, preservation of renal function, and absence of major perioperative complications [6]. The oncological equivalence of PN to RN is well documented [6]. Commonly, transient vascular occlusion is performed during PN in order to facilitate tumor excision, control bleeding, reconstruct renal parenchyma, and avoid postoperative major complications [7]. This maneuver exposes the remnant kidney to warm ischemia- (WI-) reperfusion injury [7]. In addition, the excision of the renal tumor and the inner/outer renorrhaphy are stable factors that decrease functional renal parenchyma during PN [7]. So, renal ischemia is an important, modifiable risk factor which can affect renal function permanently or temporarily for approximately 6-12 weeks postoperatively [8, 9]. WI time of 20-25 minutes is generally considered safe, while cold ischemia (by intraoperative renal cooling) can be applied if more ischemia time is demanded [6]. Our patient underwent laparoscopic PN for the left sided tumor with renal artery occlusion and WI time was 24 minutes. There were no major complications and the surgical margins were negative; however he presented great decline of eGFR which demonstrated minimum increase 40 days postoperatively.

Efforts to minimize WI time resulted to the introduction of terms like early unclamping technique, selective clamping technique, clampless PN, and selective renal parenchymal ischemia [6, 10]. Technique of selective renal parenchymal ischemia has been described by many authors who used different surgical tools or applied manual compression, which was firstly described by Semb in 1956 [10]. Selective renal parenchymal ischemia is indicated for tumors which are exophytic and are peripherally located [10]. There is no need for pedicle dissection and global renal ischemia which are technically challenging and time-consuming and may impede postoperative renal function [10, 11]. Furthermore, controlling kidney's position is easy and it allows applying different degrees of compression quickly during tumor's resection and renal reconstruction, which facilitates the procedure [10]. Following the first operation, we had to face two tumors of the contralateral kidney, one of which was sizable, and to prevent additional renal injury. Open PN with manual compression of the renal parenchyma allowed resection of two polar tumors and preservation of renal function.

There are no clear guidelines in the literature regarding the staged procedure in bilateral, synchronous RCC [5]. One opinion is to treat the larger tumor first due to concern for progression to metastatic disease [5]. In addition, tumor size is related to tumor grade, metastatic potential and cancer-specific survival and the concordance in histological subtype of bilateral, sporadic RCCs range between 70 and 99%, which were confirmed in our case [2, 5]. The second opinion is to operate the more favorable tumor first in order to preserve as much renal function as possible, before operating the more involved kidney [2]. It is well documented that chronic kidney disease is associated with increased cardiovascular morbidity and worse quality of life [2]. Regarding the time interval between staged PNs, most authors suggest time interval of approximately 2 months [8].

PN is a technically demanding and stressful procedure, especially in the case of multiple, bilateral, synchronous RCCs [1, 3]. The urologist must be experienced and ready to use all the available surgical techniques in order to achieve the optimum outcome for the patient, oncologically and regarding the renal function. Besides that, the right approach should not be planned only on surgeon's skills but should be tailored on patients' and tumors' characteristics.
